# Automatic docking of a small number of ligands into a large number of binding sites

**DOI:** 10.1186/1758-2946-5-S1-P5

**Published:** 2013-03-22

**Authors:** Alexander Kos

**Affiliations:** 1 AKos GmbH, Steinen, D-79585, Germany

## 

Very fast docking programs [1] enable new applications. In predefined workflows we start with an SDFile, filter the structures by substructure queries, followed by PASS predictions [2]. The remaining few structures are docked into 100 binding sites chosen for predicting adverse effects. The results are good indicators if a lead compound should be considered risky.
